# Neural Probes for Chronic Applications

**DOI:** 10.3390/mi7100179

**Published:** 2016-10-02

**Authors:** Geon Kook, Sung Woo Lee, Hee Chul Lee, Il-Joo Cho, Hyunjoo Jenny Lee

**Affiliations:** 1 School of Electrical Engineering, Korea Advanced Institute of Science and Technology, Daejeon 34141, Korea; geon.k@kaist.ac.kr (G.K.); lsw9403@kaist.ac.kr (S.W.L.); 2 Department of Advanced Materials Engineering, Korea Polytechnic University, Siheung 15073, Korea; eechul@kpu.ac.kr; 3 Center for BioMicrosystems, Brain Science Institute, Korea Institute of Science and Technology (KIST), Seoul 02792, Korea; ijcho@kist.re.kr

**Keywords:** biocompatibility, biocompatible coating, chronic implant, foreign body response, neural probe, neural recording

## Abstract

Developed over approximately half a century, neural probe technology is now a mature technology in terms of its fabrication technology and serves as a practical alternative to the traditional microwires for extracellular recording. Through extensive exploration of fabrication methods, structural shapes, materials, and stimulation functionalities, neural probes are now denser, more functional and reliable. Thus, applications of neural probes are not limited to extracellular recording, brain-machine interface, and deep brain stimulation, but also include a wide range of new applications such as brain mapping, restoration of neuronal functions, and investigation of brain disorders. However, the biggest limitation of the current neural probe technology is chronic reliability; neural probes that record with high fidelity in acute settings often fail to function reliably in chronic settings. While chronic viability is imperative for both clinical uses and animal experiments, achieving one is a major technological challenge due to the chronic foreign body response to the implant. Thus, this review aims to outline the factors that potentially affect chronic recording in chronological order of implantation, summarize the methods proposed to minimize each factor, and provide a performance comparison of the neural probes developed for chronic applications.

## 1. Introduction

**Figure 1:**
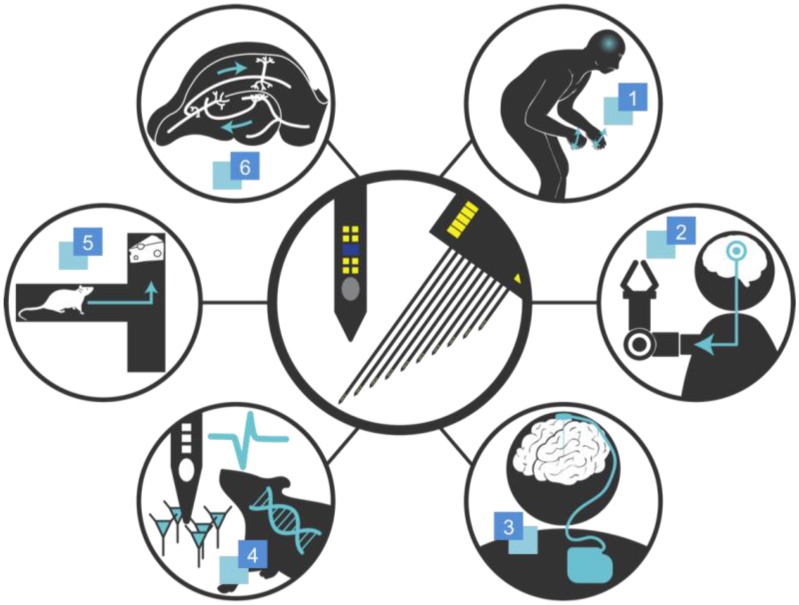
Graphical overview of applications of the neural probe technology. **1**. Parkinson’s Diseases; **2**. Neuroprosthetics; **3**. Brain Pacemaker; **4**. Investigation of Brain Diseases; **5**. Cognitive Experiments; **6**. Brain Mapping.

Recording of extracellular potentials and neuromodulation through various stimulation modalities (i.e., electrical, chemical, and optical) have a wide range of applications spanning from fundamental neuroscience research to neuroengineering for therapeutics (Figure 1). For instance, a close look at the spontaneous and stimulus-event-related signals at specific regions of the brain offers insights into the effects of brain diseases [1] and the pharmacokinetics of a drug under development [2]. Furthermore, one of the key agendas in neuroscience, which is to obtain complete functional connectome of our brain, can be addressed by observing these signals at multiple regions and correlating them to structural connectivity and behavior output [3,4]. Another important application of the recording of extracellular potentials is a brain-machine interface (BMI) developed for paralyzed patients [5,6,7,8]. The first clinical report on successful cortical control of a prosthetic arm serves as an important hallmark in the neuroprosthetics field [6]. In this study, a 3D array of 1-mm-long 96 silicon microelectrodes (i.e., 3D neural probe) was implanted in a quadriplegic to restore arm mobility. Raw neuronal signals from cortex regions that were recorded through the implanted neural probes were translated into a movement of prosthetic limbs. The patient was able to use computers by moving cursors and move a robotic arm to grasp an object. The implanted neural probe remained in the subject over a year until US Food and Drug Administration (FDA) requested removal of the probe. Another interesting application is BMI-induced plasticity which induces long-term modification of synaptic connections through recurrent stimulation [9].

**Figure 2:**
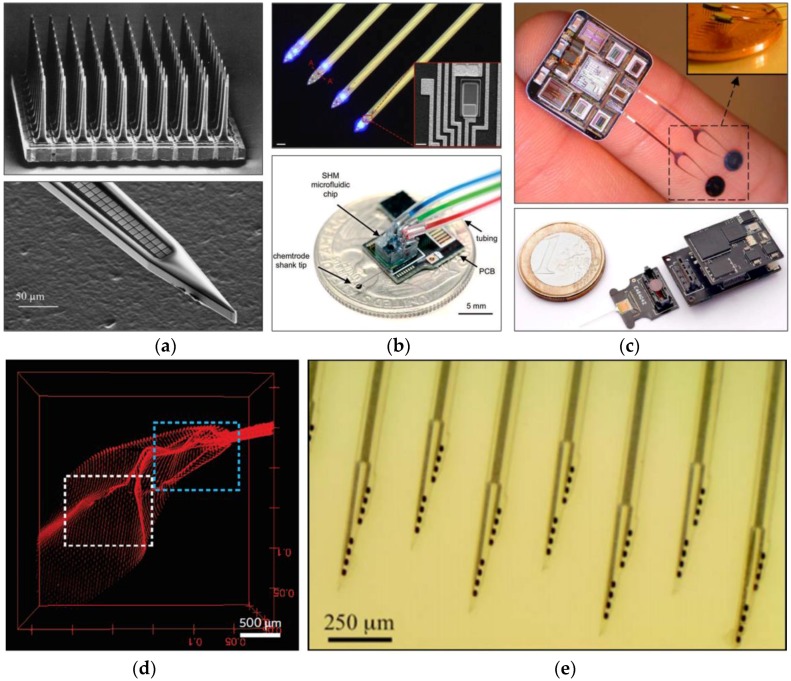
Optical photographs of the state-of-the-art neural probes: (**a**) high density (top: 3D array from Normann et al. [24]; bottom: 200-electrode shank from Scholvin et al. [17]); (**b**) functionalities (top: micro-LED for optogenetics from Wu et al. [13]; bottom: mixer-integrated microfluidic channel for drug delivery from Shin et al. [11]); (**c**) integrated circuits (top: a 64-channel integrated circuit (IC) with wireless transmission from Sodagra et al. [19]; bottom: a 52-channel IC from Lopez et al. [18]), and (**d**-**e**) biocompatibility ((**d**): syringe-injectable flexible 3D probe from Liu et al. [62]; (**e**): dissolvable silk-coated probe from Wu et al. [35]). Standard neural probes consist of single or multiple shanks and microelectrode arrays integrated at the end of the shanks for neural recording; The syringe-injectable probe shown in (**d**) is a new type of neural probe which provides 3D access to the brain with minimal damage. All figures reprinted with permission.

As the first sensing component that directly interfaces with the tissue, neural probes are important components in the neural recording system. A neural probe is an array of long microneedles (i.e., shanks) integrated with microelectrodes for extracellular recording at multiple brain regions and various functionalities for stimulation [10,11,12,13]. Since the first report on the micromachined multielectrode for extracellular recording [14], the neural probe technology has advanced on all fronts over the past half a century: density [15,16,17], functionalities [11,13], interface circuits and systems [18,19], and biocompatibility [20,21,22,23] (Figure 2). With a rapid development in microfabrication techniques, not only has the number of shanks increased from single shank to 2D [12] and 3D [24,25], but also the number of microelectrodes in a single silicon shank has dramatically increased from single to two hundred [17]. In addition to the conventional electrical stimulation, more functional features, such as microfluidic channels for drug delivery [2,10,26] and optical sources for light stimulation [13,27,28,29,30], have been integrated. Interface integrated circuits (ICs) have also advanced in terms of the number of channels, power consumption, weight, and wireless transmission [19].

Lastly, a considerable amount of work has been dedicated to optimizing the electrode-tissue interface to minimize the foreign body reaction. With rapid advancement of soft lithography and interdisciplinary research between engineering, material science and neuroscience, the materials such as biocompatible coatings [31,32,33], bioactive coatings [20,21,22,23,31,33,34,35,36,37,38,39,40], and flexible substrate materials (e.g., polyimide [41,42,43,44], parylene-C [30,45,46,47,48,49,50,51], SU-8 [52], polycarbonate [27], silk [35], and fibers [27], carbon nanotube (CNT) [53], and polyvinyl acetate [54]), have been explored. (Note that the term ‘biocompatible’ is used to imply a characteristic of a material that exhibits good biocompatibility in the central nervous system (CNS) [55].) In addition, to aid precise insertion of the flexible neural probes, several materials such as fast degrading polymer [56], Matrigel [57], and silk [35], were used to provide the mechanical support. Through this extensive exploration of fabrication methods, structural shapes, materials, and stimulation functionalities, neural probes are now denser, more functional and reliable. For more in-depth reviews on the history and development of the neural probe technology, the reader is referred to the following reviews [58,59,60,61].

However, despite this advancement, achieving chronic reliability of neural probes is still a major challenge due to the chronic foreign body response. Neural probes that record with high fidelity in acute settings often fail to function reliably or suffer from low signal amplitudes in chronic settings. The current studies on neural probes report chronic reliability in the order of weeks and few months [34,35,42,48,63,64,65,66]. Unfortunately, many neuroscience applications, such as brain mapping, investigation of brain diseases, and BMI, rely heavily on behavior studies, which require long-term reliability and viability of the recording system. For instance, neuroprosthetics application requires a reliable reading of the brain signals over a time period in the order of years to be clinically viable [67]. However, enhancing chronic reliability is challenging because the electrode-tissue interface is governed by complex biological processes around the implant and requires a multidisciplinary understanding of biology, material science, and engineering.

Thus, to resolve this important yet challenging problem of chronic recording, both research groups in the neuroscience and engineering disciplines have dedicated their efforts to understand, evade, and improve the chronic response of the electrode-tissue interface over the past decade, [20,31,33,58,64,68,69]. In addition, recently, there have been excellent in-depth reviews on various strategies developed to enhance chronic reliability [70,71,72,73,74]. However, a performance comparison of the state-of-the-art neural probes for in vivo chronic recordings has not been reported. Thus, in this review, we aim to provide the reader with snapshots of the current trends in performance. After outlining the possible factors that affect quality and duration of chronic recordings, potential solutions proposed by different groups are summarized and their efficacy in enhancing the chronic reliability are discussed in depth (Table 1).

## 2. Potential Causes of Damage

To ensure the long-term fidelity of the neural probes, it is important to examine the major modes of failure. While the foreign body reaction around the implant is considered to be the most influential factor that sets the upper limit on the period of chronic recording, there are other modes of failure that adversely affect the quality of acute extracellular recordings [68,69]. Thus, these failure modes are discussed in a chronological order of implantation of a neural probe (Table 1). From the moment of surgery, the brain is constantly exposed to external disturbances: physical damage through drilling, initial impact from penetration, physical damage and pressure build-up during the implantation, acute inflammatory response, chronic foreign body reaction, and other macromotions due to cable attachments. (Note systematic failures such as broken probe, cables, and packages are excluded).

The fidelity of acute and long-term recordings often depends on the quality of the implantation surgery [76,77]. After fixing the animal head in a stereotaxic frame and removing the scalp, craniotomy is performed using a drill at multiple locations to create openings for the probe, ground screws, and stabilizing screws. Since the thickness of a mouse skull is only around 300 μm, craniotomy performed by an inexperienced operator could damage the outermost layer of the brain and result in excision and bleeding. If the target recording area is near the surface, craniotomy must be performed with great caution. Furthermore, bleeding at the target and ground sites adversely affect the recording quality (i.e., low signal-to-noise ratio (SNR)) due to the fouling at the surface of microelectrodes and ground screws. In addition, the neural probe is often lowered using a microdrive that produces a linear motion by confining an angular motion of a knob [78]. Although minor, the friction caused from this translation results in undesired sideway movements of the neural probe which produces a dead zone larger than the actual probe size.

As the neural probe is inserted into the brain, the brain suffers from the first set of mechanical trauma that results in disruption of blood vessels, dural dimpling, and an increase in pressure around the implant [79]. While a study on the initial response of the brain to the implant showed that hemorrhages and edema comprised only 6% of the total area of the probe [80], the extension of the acute injury plays a significant role in the consequent long-term responses. The two main roles of the acute inflammatory response are to protect and restore; cytokines and neurotoxic free radicals are released in an attempt to degrade the foreign body while nearby macrophages are recruited towards the implant to remove the excess fluid and cellular debris [81]. Thus, during this acute inflammatory response, many studies show large fluctuations in the impedance measurement and neural recordings [48,64,66].

The acute inflammatory response is a transient process that diminishes over the first 6 to 8 days post-implantation [27,75,79]. For chronically implanted neural probes, activated microglia and reactive astrocytes were observed after two and four weeks post-implantation while no immunoreactivity was observed in the stab wound control experiment [79,82]. When the macrophages fail to degrade the implant, they attempt to isolate the implant by forming a dense encapsulation layer (i.e., glial scar) and also fuse into multi-nucleated “giant” cells. The glial sheath that forms as early as 6-week post-implantation becomes denser over two to four weeks and extends 50–100 μm around the insertion site. Thus, the chronic response is characterized by the presence of an elevated expression of filament proteins such as glial fibrillary acidic protein (GFAP) and vimentin, activated microglia, foreign body giant cells, and glial scar [82,83]. Furthermore, the encapsulation not only increases the impedance of the electrode-tissue interface, but also inhibits axon growth and lowers the neuronal density around the implant [83]. In addition, in chronic settings, the implant site is continuously exposed to new injuries induced by micromotion of the brain and macromotion due to cable attachment on the headset. Due to this complexity, evading the chronic response to achieve chronic reliability has been a challenging task. For more in-depth studies on the effects of brain tissue response to neural implants, the reader is referred to the following articles and reviews on biochemical pathways to the implant [70,79,84].

## 3. Common Assessment Methods of Chronic Reliability

Prior to discussing the potential solutions to achieve reliable long-term extracellular recordings, common methods used in the literature to access biocompatibility and chronic recording capability are described. Biological assays, electrochemical impedance spectroscopy (EIS), and signal-to-noise ratio (SNR) measurement are three common methodologies to assess chronic reliability and viability of the implanted neural probes. While biological assays and EIS aim to examine the immune response around the implanted neural probe and assess biocompatibility, SNR offers a direct measure of chronic reliability. The employed methods, however, vary between the disciplines of the research groups, often limited by the available experimental toolkits. Thus, it is necessary to understand these methods in depth and discuss the implication of the measured results. As the interdisciplinary research has become the common ground between neuroscience and engineering, the community should expect a more comprehensive assessment of chronic viability of neural probes including biological assays as well as EIS and SNR [27,85].

### 3.1. Biological Assays

The foreign body response is one of the key factors influencing the long-term recording of neural signals [79]. Thus, a considerable amount of work has been dedicated to observing the tissue responses to the implanted neural probe over a time course [20,31,80,81,86,87]. Currently, histological staining, assisted with immunohistochemistry (IHC) on the excised brain tissue, is the most common method to observe the foreign body response to the implanted neural probe (Table 2). After tissue perfusion, fixation, and section, thin brain slices are mounted on glass slides and are stained with multiple dyes to observe cell morphology, population, types, and location. Dyes such as Cresyl violet (or Nissl stain) that attach to nucleic acids are used to enhance the contrast to visualize and locate neurons. IHC, on the hand, provides specificity to certain cell types through the primary antibody-antigen reaction, which is then visualized through labeling using the secondary antibodies conjugated to enzymes or fluorophores. Thus, immunohistochemical staining is additionally performed to observe the foreign body reaction such as GFAP and anti-vimentin.

Within the histological studies, many variations exist in the study protocols. For example, the literature reports IHC results on the explanted neural probes [79]; on a different type of neural probes [69,88]; on different implantation duration [80,82]; and on different animals [27,80,89]. Furthermore, in addition to in vitro histological staining, biological assays such as in vivo microdialysis sampling [90] and in vitro cell culture on the neural probe [91] have been employed to examine the biocompatibility. Even within the same study, the tissue response was observed to be asymmetric around the implant and not uniform within the same layer of the brain [92]. However, in vivo and in vitro biological assays are the most direct method to examine the complex biochemical pathways that govern the tissue responses to the implants. Thus, previous studies demonstrated the evidence of mechanical trauma of insertion, short-term inflammation response, cell migration, and increase in reactive astrocytes and activated microglia around the implant, all through biological assays [80,84,88,92,93]. However, it is important to note that there is not yet a clear quantitative relationship between the degree of tissue response to the recording quality. While the proximity of the neurons near the microelectrodes and minimization of glial encapsulation were considered to be critical in achieving a good recording quality [79,82], recent studies reported contrary results: poor recordings despite the proximate neuronal cell population and reliable recordings despite heavy glial encapsulation [92].

### 3.2. Electrochemical Impedance Spectroscopy

Electrochemical impedance spectroscopy (EIS) measures the electrical impedance of the electrode-tissue interface at multiple frequencies. Because of its non-invasive nature, as opposed to that of biological assays, once the neural probes are implanted, changes in the electrode-tissue interface can be monitored over a long period of time using the same animal. In addition, EIS does not require expensive neural recording equipment or access to a biology laboratory. Thus, EIS has been a popular method, especially among the engineering groups, to indirectly evaluate the chronic performance of the newly developed neural probes.

EIS of an implanted neural probe is often performed in three-electrode cell configuration; the brain serves as the electrolyte, an implanted stainless steel or titanium bone screw as the reference electrode, and the microelectrode on the neural probe as the working electrode. An alternating current (AC) sinusoidal signal with a small amplitude in the order of mV is applied at various frequencies. The frequency range is typically 10 Hz–100 kHz, two orders of magnitude smaller and larger than 1 kHz, respectively. The impedance of the microelectrode at 1 kHz is considered biologically significant and often used as a performance metric because the width of the individual neural spike signals is approximately 1 ms [94].

Studies showed that an increase in the impedance is observed over time as a glial scar forms and insulates the implant [81,83]. Specifically, the recent study showed an increase in the impedance over the first 3 weeks of the implant due to biofouling [95]. However, several works reported that the change in the impedance does not closely correlate with the actual quality of extracellular recording [58,83]. For example, an electrode that continuously exhibited low impedance failed to record choric neural signals. Several studies showed that slow regression of neurons away from the implant is the main cause for the failure rather than the increase in impedance [83,87,95]. Here, it is important to note that a high recording quality implies either a low mean noise level or a high mean spike amplitude (or both). Thus, to gain further insight into the relationship between the impedance and the recording quality, noise level and spike signal amplitude data should be analyzed separately. A recent study that examined a large data set of spike signals within the same rat showed a weak but strong inverse relationship between the impedance and the SNR and a positive relationship between the impedance and the noise level [96]. In addition, another study showed that scaring did not affect the mean spike amplitude unless it displaced neurons away from the site-tissue interface [95]. In conclusion, a low impedance at the interface is preferred to achieve a low noise level, but is not a sufficient condition for signal detection [70,96]. Moreover, although the EIS measurement may indicate the formation of a glial scar around the implant, it does not necessarily indicate chronic viability of the neural probe and must be accompanied with other assessment methods.

### 3.3. Signal-to-Noise Ratio (SNR)

Actual recording of the neural spike signals using the implanted neural probe is the most direct method to assess the chronic recording capability. Thus, a common statistical metric, SNR, is often used to evaluate the fidelity of the chronic recording over time. A bandpass-filtered extracellular recording consists of background noise signals and distinct individual neuronal spikes. Since the ratio between these two sets of signals can be defined arbitrarily, the definition of SNR reported in the literature differs between groups. Therefore, it is difficult to use the absolute values of the SNR to compare the performance of different neural probes. Some works defined SNR as the statistical metric based on Gaussian distribution while others defined it as the ratio between the maximum signal amplitude to either twice or three times the average noise levels [64,66,69]. Recently, a new definition of SNR for a single neuron was also reported, which is based on the biophysical properties of neurons rather than a standard Gaussian distribution [97].

SNR of the recorded signals may decrease over time due to the chronic foreign body response such as insulation from the glial scar, migration of neurons away from the electrode, and lack of biocompatibility such as electrode fouling [79]. Therefore, the SNR measurements over the duration of the implantation not only indicate chronic reliability of the neural probe but also provide insights into the state of the electrode-tissue interface. In addition, since SNR is a measure of only single-unit recording characteristics, a recent work provided a new metric that accounted for both the multi-unit and local field potential (LFP) recording characteristics to evaluate the electrode performance [98]. This group also demonstrated that the electrode performance was depth (i.e., brain region) dependent urging for a more comprehensive analysis to evaluate the electrode performance [92].

## 4. Strategies to Achieve Chronic Reliability

The literature reports a large amount of work on biocompatibility and chronic reliability of many neural probes developed over decades. However, the reported chronic reliability is based on different assessment methods and different in vivo protocols (e.g., types of animals, the numbers of animals, durations of implantations, definitions of failures, and reasons for terminations). Thus, identification of trends between studies through comprehensive data analysis is challenging. However, a close examination of individual study that reports on factors that influence chronic reliability would provide us with insights into an ideal neural probe system for chronic applications. Recently, there have been excellent in-depth reviews on the strategies employed to enhance chronic reliability [70,71,72,73,74]. However, a tabulated performance comparison of the state-of-the-art neural probes for in vivo chronic recordings has not been reported. Thus, in this section, we describe various methods proposed in the literature to optimize neural probes for chronic applications in the order presented in Table 1 and aim to provide the reader with snapshots of the current trends. Since the major factor that hinders chronic recording is the foreign body response, strategies to minimize the tissue response to the implanted neural probe are most popular. Thus, these strategies are discussed in more detail compared to non-biological strategies.

### 4.1. Implantation Procedure

As the damage incurred during the insertion affects the area of kill zone around the implant, it is important to optimize the implantation procedure and to cause the minimal damage during the insertion. Since every study uses different neural probes, different animals, and different insertion methods, there is no consensus on the optimal insertion procedure. While some studies reported that fast insertion in the order of m/s is preferred because of less stiction to the neighboring tissues and less dural dimpling [80,99,100], some studies claimed that slow insertion in the order of μm/s was the key factor that led to successful recording [89]. The preferred insertion method among several options such as hand, microdrives, and pneumatic drive [101] also varied between groups. For example, insertion using a pneumatic drive was proposed to overcome the sideway movements of the probe which is often generated when inserted using a microdrive especially at a high insertion speed [101]. In addition, an integrated micromachined system composed of a microdrive and a polysilicon microelectrode array was reported for chronic recording applications; the integrated microdrive enabled fine vertical movements (in a step of 9 μm) of the implanted probe with a goal to penetrate through the glial sheath over the period of chronic recording [102]. Lastly, neurovascular mapping obtained from two-photon microscopy and endoscopy has been employed to provide a guide during the probe insertion; vascular structures were avoided using this mapping to minimize the initial BBB injury [70].

Flexible materials have been widely explored as the substrate material to reduce the damage incurred due to micromotion of the brain. One of the challenges associated with the flexible neural probes is the precise insertion of the probe without buckling or bending. Thus, stiff materials such as fast degrading polymer [56], Matrigel [57], and silk [35] that readily dissolve in the brain by proteolytic enzymes have been used to provide the mechanical support. Moreover, these supporting materials allowed for the implementation of various shapes such as the fish-bone shape [103], fork shape [35], and sheath shape [57]. By reducing the distance between the microelectrodes and the neurons, these non-standard shapes allowed for signal recording with a high SNR. Another interesting method that provides temporary mechanical support is rapid freezing of the flexible parts using liquid nitrogen [85].

### 4.2. Design of Neural Probes

While it is difficult to control and intervene with the chronic foreign body reaction, the design and fabrication of neural probes can be readily controlled. Thus, many studies have compared various design parameters such as materials [31,104], texture [44], size [69], tip geometry [89,105], and shape [85]. However, the effects of probe design on the long-term viability of neural probes differed between the reported studies. For example, a comprehensive study on the immune response to silicon implants of different sizes, surface textures, and tip geometries showed that these design factors affected the extent of acute inflammatory response but not the chronic responses such as scar formation [75]. Another study that compared different types of commercially available silicon neural probes supported this finding [58]. However, a similar study on silicon neural probes showed that a smaller probe and a cylindrically-shaped neural probe resulted in reduced glial scarring and neuronal loss [20,69,106].

Furthermore, comparative studies on the effects of design parameters are often limited to passive probes without electrodes [31,104,105] and only a small number of works included the SNR measurement data [69]. Thus, a direct relationship between the design parameters and the chronic recording quality (e.g., SNR) is difficult to identify. For example, a study showed that the mechanical mismatch between the neural probe and the brain tissue played an important role in the formation of the glial scar but the effect of this glial scar on chronic recording quality was not shown [104,107].

### 4.3. Biocompatibility

**Figure 3:**
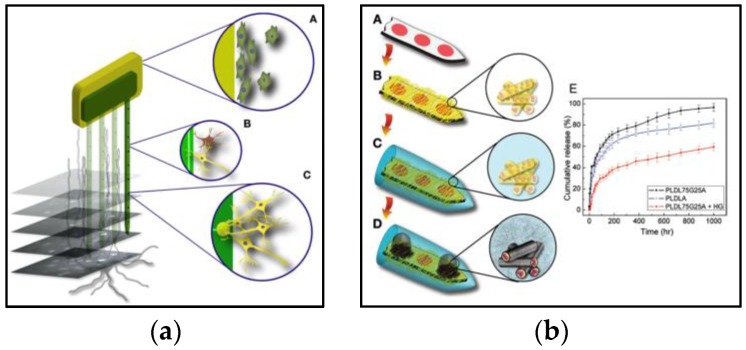
(**a**) Graphical representations of common surface modification strategies, reprinted with permission from Marin et al. [81]. An optimal surface should consist of an insulation layer that facilitates the adsorption of proteins, adhesion of fibroblast, and adhesion of neurons and glial cells without macrophage reaction and a microelectrode surface that attracts neurons without adhesion of fibroblasts or macrophage reaction; (**b**) Graphical representation of selective coatings of different bioactive materials on a neural probe, reprinted with permission from Abidian et al. [116]. Drug-loaded biodegradable nanofibers are encapsulated by a biocompatible alginate hydrogel as the insulation layer and poly(3,4-ethylenedioxythiophen) (PEDOT) is electrochemically polymerized on the microelectrode.

The most critical factor that limits the long-term chronic recording is the complex biological process that occurs around the implant: acute inflammatory response and chronic foreign body response. The extent of this biological process depends on the material of the surface of the neural probes, such as the materials of the insulation layer and electrodes. Thus, various surface modification strategies have been proposed to directly intervene with the immune response: biocompatible materials and coatings [64,83,108], bioactive coatings [83,109,110,111,112], and controlled drug release [37,40,113] (Figure 3); some of these works conducted chronic recording experiments utilizing their modification strategies and they are tabulated (Table 3).

The first attempt was to explore different materials for the insulation layer and the electrode. However, no significant improvement in terms of chronic reliability has been observed for different biocompatible metals such as iridium, stainless steel, tungsten, and platinum [61,114] and for the non-biological coating for the insulation layer [115]. Subsequently, researchers have deposited biocompatible materials such as poly(3,4-ethylenedioxythiophen) (PEDOT) on the electrodes [64,83]. The PEDOT coating resulted in a 17% increase in the number of units recorded in vivo [64] while the PEDOT coating with the synthetic peptides (DCDPGYIGSR) resulted in a decrease in impedance by 100 times [83]. The synthetic peptides were used to enhance cell adhesion. Recently, another biocompatible layer, based on a hydrogel, was evaluated and its immune response was compared to that of an uncoated polydimethylsiloxane (PDMS) neural probe. For the probe that was coated with a polyethylene glycol (PEG)—polyurethane (PU) hydrogel, an increase in the neural density and a decrease in the number of astrocytes were observed at the insertion site [108]; successful modulation of the immune response was achieved through the use of biocompatible materials. For more in-depth studies on the effects of brain tissue response to neural implants, the reader is referred to the following comprehensive reviews [71,72,73,74,79].

Another strategy to interfere with the immune response was to coat the electrode with bioactive materials. Since the high density and proximity of the neuron around the implant are crucial in unit recording, bioactive coatings that promote neuronal growth (e.g., nerve growth factor (NGF) [110]) and promote cell adhesion (e.g., laminin [109] and peptides [83]) were investigated. Furthermore, as neural processes tend to grow on astrocytes [79], bioactive coatings that attract astrocytes such as immobilized peptides (KHIFSDDSSE) [117], collagen, fibronectin, and laminin [114] were selectively coated to increase the neural density around the implant. Conversely, bioactive materials on the electrodes should prevent adhesion of glial cells. Bioactive materials such as dextran can be used for this purpose [115].

A more aggressive strategy to intervene with the immune response is to release anti-inflammatory compounds such as dexamethasone [37,118,119] and neurotropic medium [113]. The study on the dexamethasone-coated neural probe showed a 60% decrease in immunoreactivity during the first week while the number of astrocytes decreased by 50% for both the first and fourth week [119]. In addition, when observed after 46 days post-implantation, the increase in the impedance due to the encapsulation (glial scar) was smaller for the probe that released dexamethasone in a bio-degradable transporting material [37]. A new type of anti-inflammatory nanogel (polydimethylsiloxane modified N,O-carboxylic chitosan (PMSC) incorporated with oligo-proanthocyanidin (OPC)) was recently introduced [40]. A decrease in immunoreactivity by 40%–80% and an increase in the neuronal density by 100%–1100% were achieved. The study also showed an alleviated edema, reduced tissue trauma, reduced impedance, as well as an increase in signal stability. Another recent study incorporated bacterial enzyme chondroitinase ABC (chABC), which promotes recovery after spinal cord injury, into a biocompatible silk film to ameliorate axonal growth inhibition around the implant [33].

### 4.4. Flexible Substrate

Once the neural probe is implanted in our brain, the insertion site is constantly damaged due to micromotion of the brain. Micromotion of the brain is caused by changes in blood pressure induced by cardiac pulsation and breathing. A recent study showed that the density difference between the tissue and the neural probe played an important role in determining the extent of glial scarring [104]. A significantly low number of astrocytes and microglia were observed around the hollow neural probes. In addition, another group developed a finite element model to analyze the electrode-tissue microenvironment and simulate the effect of micromotion on the interfacial strains. Elevated strains were observed at both the edges of the neural probe and the tip. Since the Young’s modulus of the brain is only in the order of kPa, these studies suggest uses of soft materials for neural probes in order to minimize the mechanical impedance mismatch [59]. Another recent study demonstrated a significant reduction in the long-term chronic neuroinflammatory response by using a mechanically-compliant implant [121]. The first soft material that was explored was polyimide [41,42,43,44,63,86,99,122] which was initially used as a coating material in the early stage of deveopment. With the advent of soft lithography, polyimide is now often used as the main substrate material. The work on all-polyimide neural probes demonstrated less immunoreactivity around the implant. However, no long-term chronic recording data was provided to conclude any direct relationship between the use of polyimide to chronic reliability [63]. In fact, over the past decade, neural probes based on a vast number of soft materials such as parylene-C [30,34,45,46,47,48,49,50,51,123], polycarbonate [27], SU-8 [2], and benzocyclobutene (BCB) [124] have been reported. However, only a few of these works demonstrated the long-term neural recording (Table 4).

Due to the variability in the protocols such as type and number of animals, reasons for termination, and dimensions of the probe, no solid conclusion can be drawn on the effect of soft materials on the quality of chronic recording. However, merely comparing the demonstrated duration of the chronic recording, no distinctive advantages of soft materials have been demonstrated yet in the literature; one of the longest chronic recordings reported in the literature (–137 weeks) was achieved by the rigid silicon neural probe (Table 4). Considering that the difference between the Young’s modulus of silicon and the brain tissue (~170 GPa vs. ~3kPa), it is surprising that no flexible chronic neural probe has demonstrated a longer chronic recording capability. This is another reminder that SNR data is the most direct and reliable measure of the long-term chronic reliability as opposed to IHC and impedance data. A study showed that an upper limit for the rigid silicon neural probe for chronic recording was approximately 8–10 months post-implantation as the probes were expelled from the tissue [125]. However, there is not yet an experimental data that shows the upper limit of a flexible neural probe system.

### 4.5. Wireless Technology and Packaging

In order to perform a chronic recording, the neural probe must be packaged and secured to the head of the animal using an adhesive or dental cement [77]. This tether on the skull exerts a tangential force not only at the tip of the neural probe but also at the surface of the brain to the tip of the neural probes [107]. Thus, a flexible interconnect and printed circuit board (PCB) have been proposed [63,129]. Another strategy was to use a Teflon sheet between the implant and the dura and Gore-Tex^®^ between the dura and cranium to prevent adhesion between the dura and the arrays. Plugging and unplugging of cables from the external head stage is another source of strain exerted between the neural probe and the surrounding tissue [67]. In addition, wire connection is undesirable for clinical applications [130]. Thus, wireless transmission systems for the neural probes are continuously being developed to achieve a larger number of channel interface, higher bit rates, and low power consumption [7,19,131].

### 4.6. Other Novel Methods

**Figure 4:**
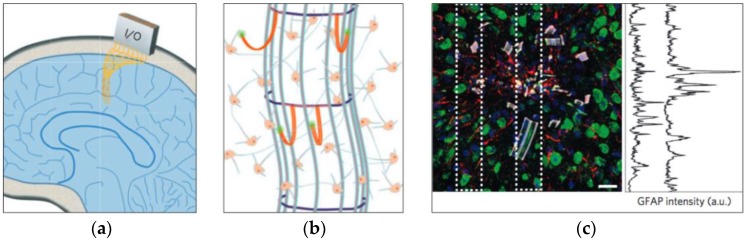
3D macroporous brain probes reprinted with permission from Xie et al. Schematics of (**a**) insertion scheme and (**b**) 3D spherical structure with flexible arms that are designed to protrude through the glial scar and form a close contact to the neurons; and (**c**) immunohistochemistry (IHC) of the insertion site demonstrating an extremely low immune response with a high neuronal density [85].

Recently, a research group has proposed a new paradigm to the neural probe technology: ultra-thin ultra-flexible 3D mesh electrodes [62,85]. These newly proposed 3D mesh electrodes address two main pressing issues in the current neural probe field: highly-dense 3D mapping of the brain and chronic recording reliability (Figure 4). By cleverly pre-straining the device and using soft lithography, microelectrodes were fabricated and integrated on thin flexible arms that extended out to penetrate through the glial scar once implanted. These legs were also integrated on a 3D mesh cylindrical structure which would enable probing of a larger area of the brain. The IHC results showed a similar neural density as well as glia density at the outer probe edge to that observed on the control sample.

## 5. Conclusions

The neural probe technology has advanced over approximately half a century. The biological processes that are involved in the electrode-tissue interface have been extensively examined and investigated to improve chronic reliability of the interface. Although the exact effects of various design factors on the long-term recording capability are still unknown, several desired features have been identified based on a large amount of studies: small dimensions, flexibility, and biocompatibility. However, the current status of the chronic performance is still in the order of weeks while many clinical applications such as neuroprosthetics require a long-term reliability in the order of years or decades. Although the maximum required period for animal studies is different from the clinical studies, at least 12 weeks of chronic recording data should be provided to fully account for the chronic immune response. With continuous innovations in the field and the strong scientific drive to understand the brain, the neural probe technology will advance in the near future to meet the stringent requirements of many interesting chronic applications.

## Figures and Tables

**Table 1:** Summary of potential causes of damages, nature of the incurred damage, and solutions to minimize the damage in chronological order of implantation *.

Stage	Before Implantation	During Implantation	Post-Implant (<1 Week)	Post-Implant (>1 Week)
**In vivo procedure**	**Acute**	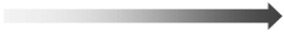		**Chronic**
	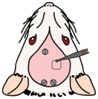	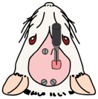	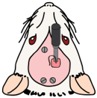	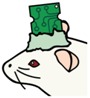
**Schematic view**	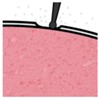	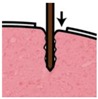	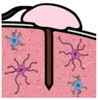	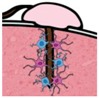
**Potential causes of damage**	Drilling of skull	Impact of insertion Mechanical vibration during implantation	Micromotion	Micromotion Macromotion Strain from cable attachment Corrosion
**Induced effects**	Undesired excision of brain tissue Contamination of electrode surface due to bleeding	Cell death Hemorrhage and edema Blood-brain barrier (BBB) disruption On-set of inflammation	Acute inflammatory response BBB disruption Unstable electrode-tissue interface	Chronic foreign body response BBB disruption Encapsulation (kill zone)
	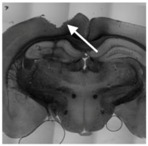	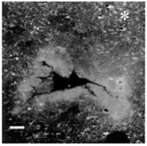	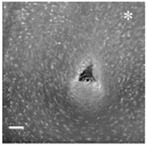	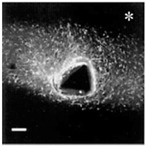
**Results**	No detection Low signal-to-noise ratio (SNR)	Larger dead zone	Unstable impedance and recording	Low SNR No detection
**Proposed solutions**	Mastery of surgery	Optimization of probe dimension Optimization of insertion speed Pneumatic insertion	Biocompatible coating Anti-inflammatory coating BBB modulation	Flexible substrate Drug coating BBB modulation Rejuvenation Wireless transmission

* Reproduced with permission from Szarowski et al. [75]. A scale bar of 100 μm.

**Table 2:** Examples of common stains and antibodies used to observe brain slices *.

Specificity	Name of Stain/Antibody	Purpose
Histology (Not cell-type-specific)	Hemotoxylin and Eosin (H & E)	Neurons (axons)
	Cresyl violet (Nissl staining)	Neurons (somata)
	Luxol Fast Blue	Myelin (fatty acid sheath surrounding axons)
Immunohistochemistry (Cell-type-specific)	Anti-NeuN ^a^, Anti-MAP-2 ^b^	Neurons
	Anti-GFAP ^c^	Astrocytes
	Anti-CD68 ^d^, Anti-ED1 ^e^, Anti-Iba1 ^f^, Anti-OX42 ^g^	Microglia/macrophages
	Anti-Neurofilament 200	Neurofilaments
	Anti-Vimentin	Vimentin (both present in astrocytes and epithelial cells of meningeal fibroblasts)
	Anti-fibronectin	Extracellular matrix (ECM) protein (up-regulated in central nervous system (CNS) scar tissue)
	Anti-IgG ^h^	BBB bleach

* Not an exhaustive list; ^a^ NeuN: Neuronal Nuclear Antigen; ^b^ MAP-2: Microtubule-Associated Protein 2; ^c^ GFAP: Glial Fibrillary Acidic Protein; ^d^ CD68: Cluster of Differentiation 68 ; ^e^ ED1: Anti-CD68 Antibody; ^f^ Iba1: Ionized Calcium-binding Adapter Molecule 1; ^g^ OX42: Anti-CD11b/c Antibody; ^h^ IgG: Immunoglobulin G.

**Table 3:** Summary of surface modification strategies.

Surface Modification	Author (Year)	Materials	Animal	Experiments
Biocompatible Coating	Cui [111] (2003)	PEDOT ^a^/DCDPGYIGSR	Guinea pig	Decrease in impedance Increase in SNR
	Ludwig [64] (2006)	PEDOT	Rat	Decrease in impedance 17% increase in number of recorded units
	Rao [108] (2012)	PEG ^b^-PU ^c^ hydrogel	Rat	Increase in neuronal density Decrease in glial density
Bioactive Coating	He [109] (2006)	Laminin	Rat	Reduction in glial density in 2 weeks No change in neuronal density
	Kim [110] (2007)	PPy ^d^/PSS ^e^/NGF ^f^ PEDOT/PBS ^g^/NGF	in vitro	22% increase in neuronal density
	Kolarcik [112] (2012)	L1	in vitro Rat	Decrease in the size of kill zone
Drug Release	Kato [113] (2006)	NGF (in microsphere)	Rat	Extension of neuritis observed
	Zhong [119] (2007)	DEX ^h^ (in nitrocellulose coating)	Rat	60% decrease in immunoreactivity 50% decrease in glial density
	Mercanzini [37] (2010)	DEX (in nanoparticles)	Rat	Decrease in impedance
	Tien [33] (2013)	chABC ^i^ (in silk film)	in vitro	Reduced glial scar
	Potter [120] (2014)	Curcumin (in poly(vinyl alcohol))	Rats	Increase in neuronal survival in 4 weeks Lost benefit of drug in 12 weeks
	Huang [40] (2015)	PMSC ^j^-OPC ^k^ (in nanogels)	Rat	Decrease in impedance Increase in SNR 40%–80% reduction in immunoreactivity 100%–1100% increase in neuronal density

^a^ PEDOT: poly(3,4-ethylenedioxythiophen); ^b^ PEG: polyethyleneglycol; ^c^ PU: polyurethane; ^d^ PPy: polypyrrole; ^e^ PSS: poly(styrenesulfonate); ^f^ NGF: nerve growth factor; ^g^ PBS: phosphate-buffered saline; ^h^ DEX: dexamethasone; ^i^ chABC: chondroitinase ABC; ^j^ PMSC: polydimethylsiloxane (PDMS) modified N,O-carboxylic chitosan; ^k^ OPC: oligo-proanthocyanidin.

**Table 4:** Summary of neural probes with chronic recording data.

Author (Year)	Neural Probe		Animal			Recording		
	Sub., Elec.	*w* (μm), *t* (μm)	Type	No.	Implant Location	Weeks	Assessment Methods	Reason for Termination
Maynard [100] (2000)	Si, n/a	n/a	Cat	8	Visual cortex	35	IHC, SNR	F
Vetter [66] (2004)	Si, Ir	55, 15	Rat	10	Auditory & motor cortex	16	IHC, SNR	S
Suner [126] (2005)	Si, Pt	40, 2.6	Monkey	3	Motor cortex	137	EIS, SNR	P
Ludwig [64] (2006)	Si, PEDOT	90, 15	Rat	8	Motor cortex	6	EIS, SNR	S
Kozai [127] (2016)	Si, PEDOT/CNT	55, n/a	Mouse	5	Visual cortex	22	EIS, SNR	S
Jackson [102] (2010)	PolySi, PolySi	50, 4	Rat	12	Cortex	20	SNR	A
Kim [48] (2013)	Parylene, Pt	350, 50	Rat	13	Motor cortex	4	IHC, EIS, SNR	S
Wu [34] (2015)	Parylene, Au	25, 65	Rat	1	Motor cortex	5	SNR	S
Lee [57] (2015)	Parylene (Matrigel), Pt	50–300, 11	Rat	2	Motor cortex	14	IHC, EIS, SNR	n/a
Cheung [63] (2007)	PI, Pt	75, 15	Rat	1	Cortex & Hippocampus	8	IHC, SNR	F
Myllymaa [128] (2009)	PI, Au	16, 5	Rat	5	Thalamus	7	EIS, SNR	n/a
Huang [40] (2015)	PI, PLGA	220, 50	Rat	10	Thalamic VPM/VPL	4	IHC, EIS, SNR	n/a
Canales [27] (2014)	PC, CPE	200, 200	Mouse	1	Cortex	8	IHC, EIS, SNR	n/a
Kozai [65] (2012)	PEG, PEDOT	8, 8	Rat	7	Motor cortex	5	IHC, SNR	S

Sub.: substrate material; Elec.: electrode material; *w*: width; *t*: thickness; PEDOT: poly(3,4-ethylene dioxythiophene); CNT: carbon nanotube; PI: polyimide; PC: polycarbonate; PLGA: poly(lactic-co-glycolic acid); CPE: conductive polyethylene; PEG: poly(ethylene glycol); IHC: immunohistochemistry; EIS: electrochemical impedance spectroscopy; A: Animal died; S: Stopped without any failure; F: No signal detected; P: Packaging issue.
